# Lab-Scale Experimental Study of Microbial Enhanced Oil Recovery on Low-Permeability Cores Using the Silicate Bacterium *Paenibacillus mucilaginosus*

**DOI:** 10.3390/microorganisms13040738

**Published:** 2025-03-25

**Authors:** Lei Li, Chunhui Zhang, Peidong Su, Hongmei Mu

**Affiliations:** 1 School of Chemical & Environmental Engineering, China University of Mining & Technology (Beijing), Beijing 100083, China; 2 State Key Joint Laboratory of Environment Simulation and Pollution Control, School of Environment, Tsinghua University, Beijing 100084, China

**Keywords:** microbial enhanced oil recovery (MEOR), silicate bacterium, *Paenibacillus mucilaginosus*, low-permeability cores, *Pseudomonas aeruginosa*, *Bacillus licheniformis*

## Abstract

Silicate bacteria, capable of decomposing silicate minerals that are widely distributed in oil reservoirs, have never been applied in microbial enhanced oil recovery (MEOR). This study investigated a typical silicate bacterium (*Paenibacillus mucilaginosus*) for the first time in a simulation experiment on low-permeability cores. Meanwhile, a biosurfactant-producing bacterium (*Pseudomonas aeruginosa*) and an acid-producing bacterium (*Bacillus licheniformis*) that have been widely studied and applied in MEOR were used for comparison. The results show that although *P. mucilaginosus* is inferior to *P. aeruginosa* and *B. licheniformis* in terms of enhancement of oil recovery at the microbial flooding stage, it can maintain efficient dissolution of minerals over extended periods during the subsequent water flooding stage. This is different from the other two bacteria and ultimately leads to a 6.9% enhancement in oil recovery (7.9% for *P. aeruginosa* and 4.8% for *B. licheniformis*). *P. mucilaginosus* improves oil recovery by increasing the porosity (1.4%) and permeability (12.3 mD) of low-permeability cores through biological weathering. The μCT results show that the pore quantity and pore volume across varying pore radii in low-permeability cores are altered after the MEOR simulation experiment by reducing the quantity and volume of pores with radii less than 10 μm and increasing the quantity and volume of pores with radii between 10 and 25 μm. Under MEOR simulation experimental conditions, *P. mucilaginosus* slightly degrade saturated hydrocarbons (1.9%), mainly the n-alkanes of C11–C20, but cannot degrade aromatic hydrocarbons, resins, and asphaltenes. The enhanced oil recovery by *P. mucilaginosus* is attributed to its bio-dissolution under neutral pH conditions, which prevents acid sensitivity damage to low-permeability cores. Thus, its MEOR characteristics are significantly different from the biosurfactant-producing bacterium *P. aeruginosa* and acid-producing bacterium *B. licheniformis*. Injecting *P. mucilaginosus* at the early stages of reservoir development or using it together with other microorganisms should maximize its MEOR effect. This study advances the MEOR framework by extending silicate-dissolving bacteria from agricultural microbial fertilizer systems to MEOR in low-permeability reservoirs, revealing the broad prospects of mineral-targeting microbes for both research and industrial applications in MEOR.

## 1. Introduction

As the exploration of medium- to high-permeability (>50 mD) oil reservoirs gradually enters the depletion stage, low-permeability (10–50 mD) oil reservoirs are becoming increasingly important worldwide, working alongside ultra-low-permeability (1–10 mD) oil reservoirs and unconventional reservoirs (<1 mD) to sustain the global oil supply [1,2,3,4]. Especially in China, the proportion of proven petroleum reserves from low-permeability reservoirs is increasing every year [5], with 46% of China’s total oil and gas resources classified as low-permeability resources [6]. Low-permeability oil reservoirs have negative characteristics that hinder development, such as low porosity, small pore throat, low-permeability, and severe reservoir damage [7,8].

As one of the most promising technologies to enhance the oil recovery of low-permeability oil reservoirs after hydraulic fracturing and initial water flooding [9], microbial enhanced oil recovery (MEOR) has been used in studies focused on multiple utilization approaches. These include non-selective activation of in situ microbial community without prior taxonomic identification [4,5,8], biosurfactant-producing bacteria (*Bacillus velezensis*, *Pseudomonas aeruginosa,* and *Bacillus subtilis*) [10,11], genetically engineered bacteria (*Escherichia coli* and *Pseudomonas* sp.) [12], and biogas-producing microcosms [13]. Due to the consideration that small pores in low-permeability reservoirs hinder the passage of microbial cells, some studies have utilized extracted biosurfactants produced by microorganisms (*B*. *subtilis* and *Bacillus mojavensis*) to enhance oil recovery [9,14]. These studies still follow the approach of MEOR in medium- to high-permeability reservoirs, with the goal of improving oil displacement efficiency [15,16]. However, the strategy of improving oil displacement efficiency proves insufficient in low-permeability reservoirs [17]. Two primary reasons account for this limitation. First, the limited pore space of low-permeability reservoirs restricts microbial biomass, resulting in low concentrations of metabolites (e.g., biosurfactants, bio-acids, biogas, and others) that are critical for enhancing oil displacement efficiency [18]. Secondly, severe reservoir damage in low-permeability oil reservoirs causes corner-bound residual oil to face significantly higher viscous resistance [19]. Consequently, scholars have initiated research on microbial-mediated flow channel improvement in low-permeability reservoirs to boost oil recovery efficiency. In this direction, attempts have involved using Fe(III)-reducing bacteria (*Proteus hauserifective*) to inhibit clay swelling in low-permeability reservoirs to reduce reservoir damage [20] and using the combination of *B*. *subtilis* and polymers to seal cracks in low-permeability reservoirs [21]. Although some attempts utilized acid-producing bacteria (*Bacillus licheniformis*) to enhance porosity and permeability in low-permeability oil reservoirs through acid dissolution [22], the outcomes were unsatisfactory. This is attributed to the acid sensitivity of low-permeability reservoirs, which can lead to reductions in both porosity and permeability [23]. The positive effects of acid dissolution are offset by the adverse impacts of acid sensitivity, resulting in minimal overall improvement.

However, silicate bacteria with strong decomposition ability in a neutral pH environment for silicate minerals [24,25,26,27], which are commonly found in oil reservoirs in the form of feldspar, clay, quartz, and mica [28], have never been studied in the field of MEOR for low-permeability oil reservoirs. *Paenibacillus mucilaginosus* is recognized as one of the most extensively studied and applied silicate bacteria due to its enzymatic dissolution of silicate minerals to release silicon and potassium ions, coupled with the production of biofilm-associated extracellular polymeric substances that enhance soil microaggregate formation and crop stress resistance, which collectively promote agricultural production [29,30,31]. Its ability to dissolve silicate minerals [32,33] makes it very promising in improving porosity and permeability to enhance oil recovery in low-permeability oil reservoirs.

For the first time, this study conducts a MEOR simulation experiment on low-permeability cores using the silicate bacterium *P. mucilaginosus*. On this basis, a biosurfactant-producing bacterium (*P*. *aeruginosa* [34,35,36]) and an acid-producing bacterium (*B*. *licheniformis* [22,37]) that have been widely studied and applied in MEOR were used for comparison. This study specifically assessed *P. aeruginosa*’s retention capacity in low-permeability cores, dissolution effects on core minerals, crude oil degradation efficiency, and potential for enhancing oil recovery.

## 2. Materials and Methods

### 2.1. Materials

#### 2.1.1. Microorganism Strains

The *P. mucilaginosus* strain CICC20666, *P. aeruginosa* strain CICC10204, and *B. licheniformis* strain CICC21886 used in this study were purchased from China Center of Industrial Culture Collection (https://sales.china-cicc.org/, accessed on 25 February 2025). All three strains are aerobic. The microbial strains were maintained as lyophilized powder in penicillin vials under cryogenic storage (Thermo Fisher Scientific, Waltham, MA, USA) at −30 °C.

#### 2.1.2. Cores

The nine low-permeability artificial cores used in this study were purchased from Hai’an Huacheng Scientific Research Instrument Co., Ltd., Nantong, China. These nine artificial cores were fabricated in a single production batch, with porosity of 15.93–17.69% and permeability of 33.0–37.3 mD (Table S1). The cores are mainly composed of feldspar (31.1%), quartz (22.6%), mica (18.2%), clay (2.9%), dolomite (2.8%), calcite (2.1%), and epoxy resin (17.0%).

#### 2.1.3. Crude Oil

The crude oil (Sample number X213W585) was collected from production well 58-5 in Xin214 Block of Jilin Oilfield, Jilin, China, with a density of 0.81 g/cm^3^ (28 °C) [38] and a viscosity of 2.34 mPa·s (20 °C) [39].

All the chemical agents were of analytical grade and supplied by Tianjin Fuchen Chemical Reagents Factory, Tianjin, China.

### 2.2. Methods

#### 2.2.1. Microbial Cultivation

The culture medium of *P. mucilaginosus* strain CICC20666 contains tryptone (15 g/L), soybean peptone (5 g/L), and NaCl (5 g/L). The culture medium of *P. aeruginosa* strain CICC10204 and *B. licheniformis* strain CICC21886 contains peptone (5 g/L), beef powder (3 g/L), and NaCl (5 g/L). The culture medium formulas for these three strains were obtained from the website of China Center of Industrial Culture Collection (https://sales.china-cicc.org/cicc/detail2/?sid=2835, accessed on 25 February 2025). The culture media were autoclaved at a GR85DR sterilizer (Zealway, Wilmington, DE, USA) for 20 min (121 °C, 1 atm) [40] before use. In an aerobic and sterile environment, the reactivation of strains was achieved by adding 1 mL of lyophilized powder to 4 mL of corresponding culture medium and incubating in a MaxQ™ 6000 shaker (Thermo Fisher Scientific, Waltham, MA, USA) for 2 days (120 r/min, 30 °C). Then, 4 mL of the culture was mixed with 40 mL of corresponding culture medium for culturing until the concentration of cells reached 10^8^ cell/mL (Figure S1) in a MaxQ™ 6000 shaker (Thermo Fisher Scientific, Waltham, MA, USA) for injection (120 r/min, 30 °C). The time required for the three microbial species to reach a cell concentration of 10^8^ cell/mL exhibited significant variation. Multiple pre-trial experiments demonstrated that *P*. *mucilaginosus*, *P*. *aeruginosa*, and *B*. *licheniformis* required approximately 36 h, 22 h, and 26 h, respectively. The pH values of the culture media for *P. mucilaginosus*, *P. aeruginosa*, and *B. licheniformis* were 6.3, 7.1, and 4.2, respectively (Table S2). The microbial cell concentration, pH, redox potential, conductivity, and surface tension of the three culture media were measured using the following methods.

#### 2.2.2. Measurement of Microbial Cell Concentration

An Eclipse Ni-U phase contrast microscope (Nikon, Tokyo, Japan) and blood cell counting plate were used to determine the concentration of microbial cells [40,41].

#### 2.2.3. Measurements of pH, Redox Potential, and Conductivity

The pH, redox potential, and conductivity were measured using three probes (Inlab Expert Pro pH, Inlab Redox, and Inlab 731, respectively) on a SevenMulti™ instrument (Mettler Toledo, Columbus, OH, USA) [41].

#### 2.2.4. Measurement of Surface Tension

The surface tension of water was measured using a BZY-1 surface tension meter (Hengping, Shanghai, China). The Wilhelmy plate method was used to measure the maximum pulling force when the bottom edge was parallel to the interface and just contacting the water [42].

#### 2.2.5. MEOR Simulation Experiment

The MEOR simulation experiment of this study was conducted on a core displacement device [43,44,45] (XIIC-YXLD, Figure S2) provided by Shengli Oilfield, Shandong, China. The whole experiment was conducted at 30.0 °C, and each bacterium was tested with three cores to offset errors (Table S1). The main experimental stages are listed as follows.

① Saturation of core with deionized water [44,45]. The core displacement device was set to achieve a back pressure of 10 MPa, a ring pressure of 12 MPa, and a flow rate of 0.05 mL/min for deionized water. The core was then flooded with deionized water 24 h to ensure its complete saturation. This study used deionized water instead of strata water as used in other studies [9] to avoid interference during elemental analysis. The pore volume (PV) was equal to the volume of deionized water stuck in the core. The porosity and permeability of core were calculated using the following equations [44,45]:

(1) $$\phi =\frac{PV}{\frac{\pi d^{2}L}{4}}$$

(2) $$\kappa =\frac{Q\mu L}{A\Delta P}$$

where ϕ is porosity of core, *PV* is pore volume (cm^3^), d is diameter of core (cm), L is length of core (cm), κ is the absolute permeability of core (D, 1 D = 1000 mD), *Q* is volumetric flow rate (cm^3^/s), *A* is area of core cross section (cm^2^), *μ* is the viscosity (mPa·s), and ΔP is the pressure drop of the fluid along the length *L* (atm, 0.101 MPa).

② Saturation of the core with crude oil [43,46]. Before starting, the crude oil and core saturated with deionized water were preheated at 60 °C for 2 h. Then, crude oil was pressed into the core at flow rate of 0.05 mL/min (back pressure is 10 MPa, ring pressure is 12 MPa) to ensure its sufficient saturation. The injection of crude oil was stopped after no deionized water was detected at the core outlet for 2 h. This was followed by aging for 7 days [47,48]. As crude oil was inserted into the core, the deionized water was displaced and discharged from the outlet end of the core. The volume of crude oil injected from the inlet end ($V_{oi}$) and the volume of crude oil flowing out from the outlet end ($V_{oo}$) were measured.

③ Initial water flooding [43]. In the initial water flooding stage, the deionized water was injected into the core at 0.05 mL/min (back pressure is 8 MPa, ring pressure is 10 MPa) until the water cut reached at least 99%. To determine a unified initial water flooding duration for all cores, we conducted multiple preliminary experiments, finding that 120 h ensures a water cut exceeding 99%. The displaced crude oil and water were collected to calculate the oil recovery of initial water flooding using the following equation [43]:

(3) $$\;\mathrm{IWFR}=\frac{V_{iwfo}}{V_{oi}-V_{oo}}100\%$$

where IWFR is recovery of initial water flooding (%), $V_{iwfo}$ is volume of crude oil collected during initial water flooding (cm^3^), $V_{oi}$ is the volume of crude oil injected from the import end (cm^3^) in Stage ②, and $V_{oo}$ is the volume of crude oil flowing out from the outlet end (cm^3^) in Stage ②.

④ Microbial flooding [43]. In this stage, the microbial culture media mentioned in Section 2.2.1 were injected into the cores. Approximately 2 PVs of microbial culture media were used for flooding at a rate of 0.05 mL/min (back pressure is 8 MPa, ring pressure is 10 MPa). As the PV values of each core are not equal, to ensure a consistent flooding duration, we used the highest PV value among the nine cores (PV_max_ = 10 cm^3^, Table S1) to calculate that the time of microbial flooding is 400 min. The enhanced oil recovery of the microbial flooding stage was calculated using the following equation [43]:

(4) $$\mathrm{MFR}=\frac{V_{mfo}}{V_{oi}-V_{oo}}100\%$$

where MFR is the enhanced oil recovery of microbial flooding (%), and $V_{mfo}$ is volume of crude oil collected during microbial flooding (cm^3^). See Equation (3) for $V_{oi}$ and $V_{oo}$.

⑤ Water flooding [43]. After microbial flooding, the deionized water was injected into the core at a rate of 0.05 mL/min (back pressure is 8 MPa, ring pressure is 10 MPa) until the water cut reached 99%. Preliminary experiments have shown that 120 h can guarantee this result for this batch of cores. The enhanced oil recovery of water flooding after microbial flooding was calculated using the following equation [43]:

(5) $$\mathrm{WFR}=\frac{V_{wfo}}{V_{oi}-V_{oo}}100\%$$

where WFR is the enhanced oil recovery of water flooding (%), and $V_{wfo}$ is volume of crude oil collected during water flooding stage (cm^3^). See Equation (3) for $V_{oi}$ and $V_{oo}$.

Normally, the crude oil displaced during microbial flooding stage and subsequent water flooding stage is attributed to microbial contributions. Therefore, the microbial enhanced oil recovery ratio is calculated using the following equation [43]:

MR = MFR + WFR(6)

where MR is ratio of microbial enhanced oil recovery (%). See Equation (4) for MFR and Equation (5) for WFR.

The water at the outlet end of core from Stage ④ and ⑤ was collected every 10 h. For water, measure the cell concentration, pH value, redox potential, conductivity, surface tension, and element concentration. Upon completion of the experiment, all collected crude oil samples undergo fractions analysis and gas chromatography-mass spectrometry analysis.

After Stage ⑤, scanning electron microscopic and energy dispersive X-ray spectroscopic analyses are performed using the core. Then, the core is soaked in dichloromethane for 24 h [49] and then displaced with a mixture of petroleum ether and dichloromethane (1:1) at a rate of 1 mL/min (back pressure is 3 MPa, ring pressure is 5 MPa) in the XIIC-YXLD core displacement device for 8 h. Then, μCT detection for the core is performed after being placed in a 160 °C oven for 24 h, and the porosity and permeability are measured.

It is noteworthy that employing low-permeability core samples to represent reservoirs in simulation experiments exhibits limitations, due to the inability to authentically reconstruct petrophysical properties, flow field characteristics, dimensional scaling, development dynamics, and historical operations (particularly hydraulic fracturing).

#### 2.2.6. Measurement of Element Concentration

The element concentrations of silicon, aluminum, manganese, and iron were assessed using the standard method [50] with an ICAP-Q inductively coupled plasma mass spectrometer (Thermo Fisher Scientific, Waltham, MA, USA).

#### 2.2.7. Fraction Analysis of Crude Oil

Fraction (saturated hydrocarbons, aromatic hydrocarbons, resins, and asphaltenes; SARA) analysis separates crude oil components according to their polarizability and polarity by column chromatography [51]. N-hexane and absorbent cotton were used to filter out asphaltenes [52]. The saturated hydrocarbons, aromatic hydrocarbons, and resins were obtained using a chromatographic column (gel and activated alumina) with n-hexane, dichloromethane, and ethanol [52].

#### 2.2.8. Gas Chromatography-Mass Spectrometry (GC-MS) Analysis of Saturated and Aromatic Hydrocarbons

The compounds in the saturated hydrocarbons and aromatic hydrocarbons of the crude oil were characterized and quantified using GC-MS [53]. Deuterated tetracosane (D50-nC24, 10 μg) and deuterated dibenzothiophene (D8-dibenzthiophene, 10 μg) were used as internal standards for saturated hydrocarbons and aromatic hydrocarbons, respectively [54]. Trace-DSQ mass spectrometer (Thermo Finnigan, San Jose, CA, USA) coupled to an HP 6890 gas chromatograph (Agilent, Santa Clara, CA, USA) was used for GC-MS analysis. The column was HP-5MS, and the carrier gas was helium (99.99%). The oven temperature of the gas chromatograph was initially set to 50 °C; was subsequently increased to 120 °C at a rate of 20 °C/min, 250 °C at a rate of 4 °C/min, and 310 °C at a rate of 3 °C/min; and was maintained for 30 min. The mass spectrometer operated in full-scan electron impact mode with an electron energy of 70 eV.

The content of each compound in the saturated hydrocarbons and aromatic hydrocarbons was obtained using GC-MS [55] and calculated using the following equations:

(7) $$C=\eta \frac{S_{a}m^{*}}{S^{*}m_{a}}$$

where C is the content of compounds in the saturated hydrocarbons and aromatic hydrocarbons (μg/g), η is the response coefficient, $S_{a}$ is the peak area of each compound in saturated hydrocarbons and aromatic hydrocarbons, $S^{*}$ is the peak area of the internal standard, $m^{*}$ is the mass of the internal standard (10 μg), and $m_{a}$ is the mass of heavy oil used in fractionation (g).

#### 2.2.9. Scanning Electron Microscope and Energy Dispersive X-Ray Spectroscopy (SEM-EDX)

The inlet end, middle section, and outlet end of the core were sprayed with gold and scanned using a VEGA3 scanning electron microscope (TESCAN, Brno, Czech Republic) [56]. The acceleration voltage of electronic detector was 20.0 kV.

#### 2.2.10. μCT Detection

A nanoVoxel3502E computerized tomography system (ZEISS, Oberkochen, Baden-Württemberg, Germany) was used to detect low-permeability cores [57]. The software Avizo (Thermo Fisher Scientific-Avizo, Merignac, France) was used for 3D digital core image processing (Figure S3) [58,59,60].

## 3. Results and Discussion

### 3.1. Initial Water Flooding Stage

The primary purpose of the initial water flooding stage is to simulate the post-development environmental conditions [61] of low-permeability cores. After initial water flooding, potential changes in the cores may include particle migration, variations in pore-throat dimensions, swelling of clay minerals [62], wettability [63], and crude oil distribution. During the initial water flooding, low-permeability cores are more prone to permeability damage caused by improper management of water [64], acid, salts, alkalis [65], velocity of flow [23], and pressure gradients [66]. This exemplifies the core conditions confronted by MEOR.

**Figure 1:**
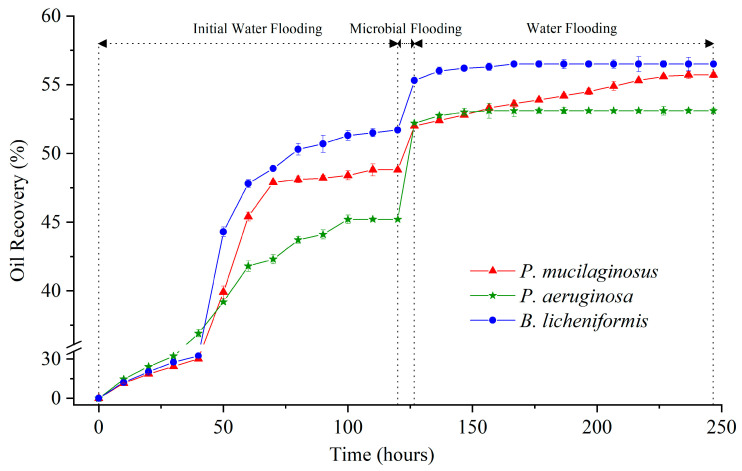
Crude oil recovery ratio of three experimental groups (*P. mucilaginosus*, *P. aeruginosa,* and *B. licheniformis*) at three stages (initial water flooding, microbial flooding, and water flooding) during the simulation experiment.

The maximum IWFR largely depends on the initial conditions of the core (Table S1), such as porosity, permeability, pore connectivity, and original oil saturation [66]. Therefore, it can be observed that the IWFR maximum of the three experimental groups differ (Figure 1), and this discrepancy persists even when using cores from the same batch and injecting identical deionized water. Such natural variation is not the primary focus of this study, and it will be excluded from the discussion of MEOR.

**Figure 2:**
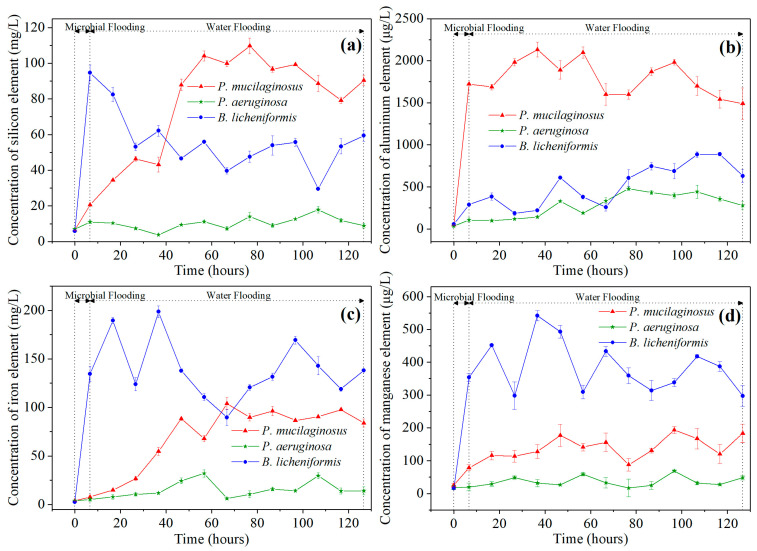
The element concentration of water at the outlet end of cores at two stages (microbial flooding and water flooding) during the simulation experiment. Note: (**a**) Silicon element, (**b**) Aluminum element, (**c**) Iron element, and (**d**) Manganese element.

During this stage, slight mineral dissolution by deionized water was also observed. This is evidenced by the absence of silicon (Si), aluminum (Al), iron (Fe), and manganese (Mn) in deionized water at the inlet end of core, while non-zero concentrations of these four ions are detected at the outlet end at the beginning of microbial flooding (Figure 2). In fact, the phenomenon of mineral dissolution is relatively common during the water flooding development process in oilfields [67,68].

### 3.2. Microbial Flooding Stage

**Figure 3:**
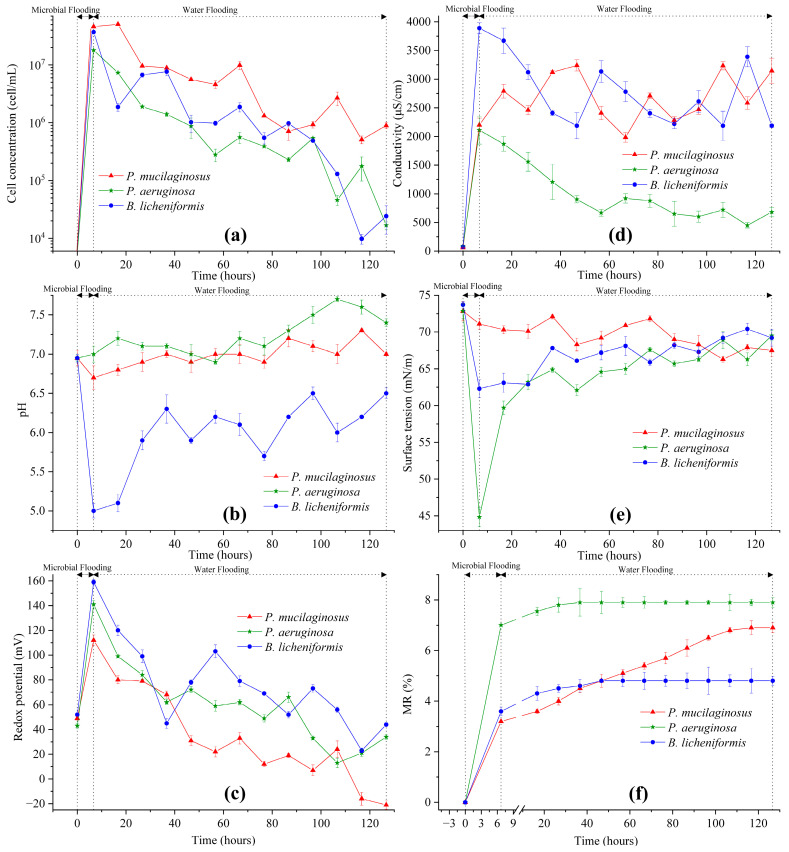
The properties of water at the outlet end of cores and the microbial enhanced oil recovery at two stages (microbial flooding and water flooding) during the simulation experiment. Note: (**a**) Microbial cell concentration, (**b**) pH, (**c**) Redox potential, (**d**) Conductivity, (**e**) Surface tension, and (**f**) Microbial enhanced oil recovery ratio. See Equation (6) for MR.

**Figure 4:**
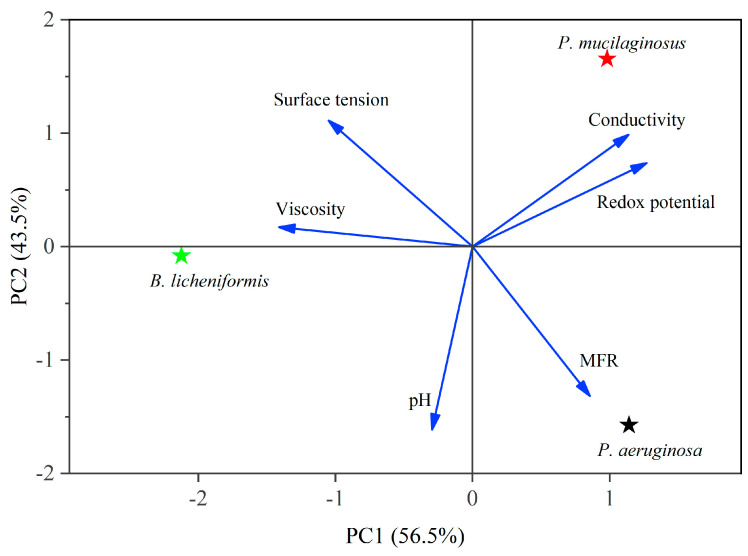
Principal component analysis (PCA) of culture media properties at the microbial flooding stage. Note: See Equation (4) for MFR.

**Figure 5:**
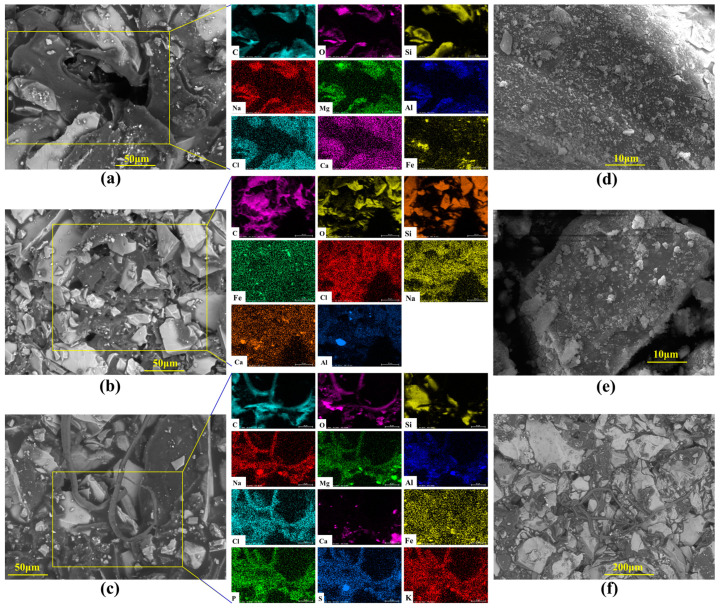
SEM and EDX images of cores after the simulation experiment. Note: (**a**) At the inlet end of the core, crude oil covers the surface, enters through pores, and is distributed between mineral particles; (**b**) At the cross-section of the core, residual oil is distributed in the corner region of the pores, and there are flowable channels; (**c**,**f**) At the outlet end of the core, crude oil is squeezed out in strips through the pores; (**d**) Inside the core, the surface of mineral particles cracks and falls off; (**e**) Inside the core, there are cracks and microbial cell debris on the surface of mineral particles.

In the microbial flooding stage, the MFR of the three microorganisms rapidly increased and reached their maximum values (3.2% for *P. mucilaginosus*, 7.0% for *P. aeruginosa*, and 3.6% for *B. licheniformis*) within a short period (Figure 3f). Due to the limited time of this stage (400 min), microorganisms have insufficient time to exert the MEOR effect by significantly impacting minerals or crude oil. Therefore, the acquisition of MFR is predominantly governed by the initial properties of the culture medium (Table S2). Principal component analysis (PCA) shows that at the stage of microbial flooding, only surface tension and MFR are strongly negatively correlated (Figure 4). This result is consistent with some research findings [35] and our observation that the *P. aeruginosa* culture medium exhibiting the lowest surface tension (Table S2) achieved the maximum MFR (Figure 3f). For the *B. licheniformis* group, the acquisition of MFR is primarily attributed to metabolically produced bio-acids [22,37] (acid-etched core) and biosurfactants [69] (enhancing oil displacement). However, due to its suboptimal biosurfactant production capacity compared to *P. aeruginosa* (Table S2), its MFR cannot rival that of *P. aeruginosa* (Figure 3f). *P. mucilaginosus* cannot produce biosurfactants or bio-acids [29]. In the microbial flooding stage, MFR is mainly obtained by the improvement of the oil to water flow ratio, which is achieved by its culture medium with a relatively high viscosity (Table S2). The scraping and carrying effects of microbial cells on crude oil are not only found in research reports [70]. They were also observed in this experiment. Specifically, microorganism-specific elements were detected in the crude oil at the core outlet (Figure 5c,f).

Microbial-induced mineral dissolution in cores has been detected in the microbial flooding stage (Figure 2). Clearly, the pH of the culture medium is one of the key influencing factors for mineral dissolution at this stage, as demonstrated by the fastest increase in the elemental concentration of the *B. licheniformis* group, which coincides with its lowest pH (Figure 2, Table S2, and Figure 3b). Additionally, the neutral pH culture medium of *P. mucilaginosus*, containing exopoly saccharides (EPSs), also promotes rapid dissolution of mineral elements [71]. In contrast, the low surface tension characteristic of the *P. aeruginosa* group contributes minimally to mineral element dissolution (Figure 2, Table S2, and Figure 3b). Although this microbial-induced mineral dissolution process cannot directly enhance the oil recovery ratio within the short-term operation period of this stage, it exhibited sustained persistence throughout the experimental duration (Figure 2) and indirectly facilitate microbial survival, which benefits the ultimate oil recovery ratio.

### 3.3. Water Flooding Stage

#### 3.3.1. Microorganisms

Following microbial cell injection into the core, a portion becomes retained within the core (Figure 5e), while the remainder is discharged from the outlet end. Of the discharged cells, some are detected in water (Figure 3a) and others in crude oil (Figure 5c). During the water flooding stage when microbial culture medium injection ceases at the inlet end, the microbial cell concentration detected in outlet water reflects microbial proliferation within the core. Microbial cell concentrations in the cores demonstrate a progressive decline (Figure 3a), correlating with decreasing trends in metabolic characteristic parameters, including redox potential (Figure 3c) and conductivity (Figure 3d). This phenomenon arises from the microbial metabolic consumption of oxidants and conductive particles. Given that the XIIC-YXLD core displacement device only allows dissolved oxygen replenishment from deionized water without alternative oxygen sources, intensified metabolic activity inevitably drives the redox potential reduction. Conductive particles are replenishable through mineral dissolution, rendering conductivity a direct indicator of the mineral dissolution extent. The diminishing cell concentration consequently reduces acidic substances and biosurfactant production, which manifest as elevated pH and surface tension (Figure 3b,e). For *P. aeruginosa* and *B. licheniformis* whose MEOR mechanisms rely on these metabolites, such attenuation leads to diminished MEOR efficacy (Figure 3f). *P. mucilaginosus* exhibited the highest retained biomass within the cores among the three microbial species (Figure 3a), despite demonstrating an overall declining retention trend over time. Although *P. mucilaginosus* underperforms in pH and surface tension compared to the other two, it demonstrates the highest biomass retention capacity in the cores. This observation is further validated by redox potential (Figure 3c) and conductivity (Figure 3d) measurements.

#### 3.3.2. Microbial Dissolution of Core Minerals

**Figure 6:**
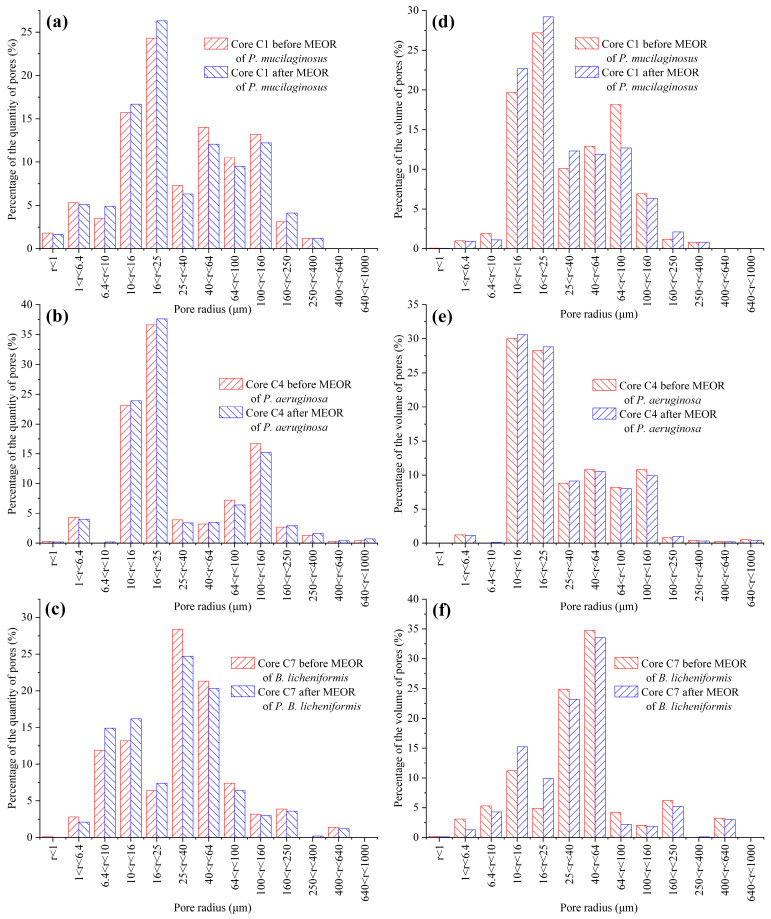
The quantity and volume distribution of pores with different pore sizes in the *P. mucilaginosus*, *P. aeruginosa*, and *B. licheniformis* groups before and after the simulation experiment. Note: (**a**) The percentage distribution based on the quantity of pores within different radius ranges in Core C1 before and after MEOR with *P. mucilaginosus*, (**b**) The percentage distribution based on the quantity of pores within different radius ranges in Core C4 before and after MEOR with *P. aeruginosa*, (**c**) The percentage distribution based on the quantity of pores within different radius ranges in Core C7 before and after MEOR with *B. licheniformis*, (**d**) The percentage distribution based on the volume of pores within different radius ranges in Core C1 before and after MEOR with *P. mucilaginosus*, (**e**) The percentage distribution based on the volume of pores within different radius ranges in Core C4 before and after MEOR with *P. aeruginosa*, and (**f**) The percentage distribution based on the volume of pores within different radius ranges in Core C7 before and after MEOR with *B. licheniformis*.

**Figure 7:**
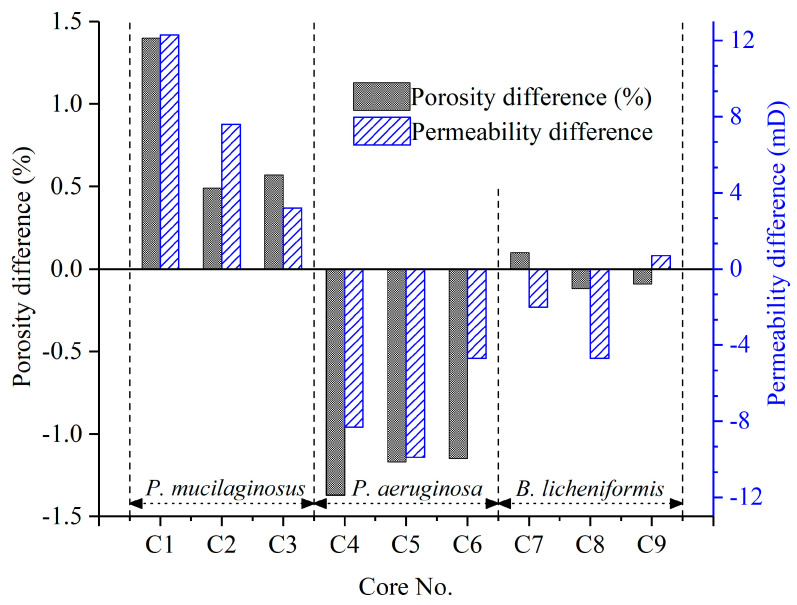
Changes in the porosity and permeability of all nine low-permeability cores after the simulation experiment. Note: The data were obtained using the core saturation method described in Stage ①.

The microbial dissolution of the core minerals was observed during the water flooding stage (Figure 2). Microscopically, microbial-induced cracking and exfoliation on mineral particle surfaces were captured (Figure 5d,e), while μCT imaging (Figure S3) documented changes in pore quantity and pore volume across different core radii (Figure 6). Macroscopically, concentrations of four elements (Si, Al, Fe, and Mn) under three microbial treatments showed significant increases (Figure 2), with conductive particles maintaining elevated levels despite no replenishment and ongoing consumption (Figure 3d). In the absence of fresh culture medium injection, this phenomenon is unequivocally attributed to microbial metabolic activity rather than culture medium properties. Two key outcomes of microbial mineral dissolution were quantified. First, the porosity of the cores changed. Both μCT data (Figure 6 and Table 1) and the core saturation method (Stage ①, Figure 7) demonstrated consistent trends. The μCT data revealed that the alterations in pore volume induced by *P. mucilaginosus*, *P. aeruginosa*, and *B. licheniformis* were 4.33%, −2.74%, and 0.85%, respectively (Table 1). Core saturation measurements showed that the maximum porosity changes in the *P. mucilaginosus*, *P. aeruginosa*, and *B. licheniformis* groups were 1.4%, −1.37%, and −0.12%, respectively (Figure 7). Meanwhile, variations in pore quantity and pore volume were observed across different pore size ranges. The pore quantity and pore volume for pores larger than 10 μm are decreasing, whereas those for pores between 10 μm and 25 μm are increasing (Figure 6). Second, the permeability of the core has changed. The core saturation method results indicated that the maximum permeability changes for the *P. mucilaginosus*, *P. aeruginosa*, and *B. licheniformis* groups were 12.3 mD, −9.9 mD, and −4.7 mD, respectively (Figure 7).

Usually, flooding causes significant damage to low-permeability cores, resulting in a significant decrease in porosity and permeability [72]. Therefore, the reservoir damage observed in the *P. aeruginosa* group is considered a typical response in low-permeability cores [73]. Achieving near-complete damage mitigation, as demonstrated by the *B. licheniformis* group, already represents breakthrough significance. The porosity and permeability enhancement exhibited by *P. mucilaginosus* under identical conditions is rare, which unequivocally highlights its superior mineral dissolution capabilities.

#### 3.3.3. Microbial Degradation of Crude Oil

**Figure 8:**
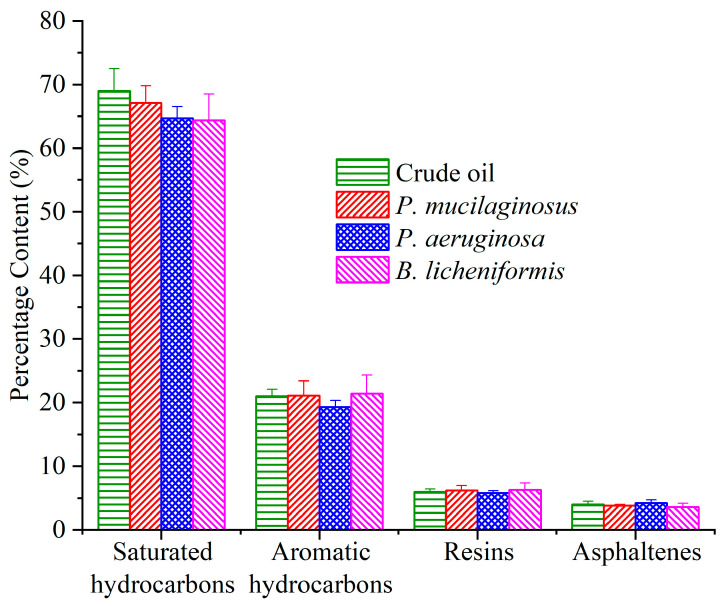
The content of the fraction components of crude oil in the *P. mucilaginosus*, *P. aeruginosa*, and *B. licheniformis* groups after the simulation experiment.

Due to the prolonged duration of the water flooding stage, microbial degradation of crude oil has also been documented (Figure 8). Following the termination of culture medium injection, the residual culture medium retained within the core was progressively displaced by deionized water. Under carbon source deprivation, the three microorganisms shifted metabolic activity to initiate crude oil degradation [37,74,75]. The results of fraction analysis of the crude oil show that all three microorganisms slightly degraded saturated hydrocarbon (1.9%, 4.3%, and 4.6% for *P. mucilaginosus*, *P. aeruginosa*, and *B. licheniformis*), and *P. aeruginosa* slightly degraded aromatic hydrocarbons (1.7%). However, resins and asphaltenes did not undergo significant degradation (Figure 8). The total ion currents of saturated hydrocarbons and aromatic hydrocarbons support this conclusion (Figures S4 and S5). Further integration of GC-MS data showed that *P. mucilaginosus* degraded normal alkanes of C10–C16 (Figure S6). *P. aeruginosa* degraded normal alkanes of C20–C39 (Figure S6), aromatic hydrocarbons of the naphthalene series with two fused aromatic rings, and aromatic hydrocarbons of biphenyl derivatives containing two aromatic rings (Figure S7). *B. licheniformis* degraded normal alkanes of C26–C38 (Figure S6).

#### 3.3.4. The Enhanced Oil Recovery of Water Flooding

During the water flooding stage, the maximum WFRs for *P. mucilaginosus*, *P. aeruginosa*, and *B. licheniformis* are 3.7%, 0.9%, and 1.2%, respectively (Figure 3f). The time required to reach the maximum WFR was 100 h for the *P. mucilaginosus* group, 30 h for the *P. aeruginosa* group, and 40 h for the *B. licheniformis* group (Figure 3f). This demonstrates that *P. mucilaginosus*, with the longest operational duration, achieved the highest WFR. This is attributed to its significantly higher biomass compared to the other two (Figure 3a) and its superior ability to dissolve core minerals. Notably, this enhanced dissolution capability was independent of acidic environmental conditions (Figure 3b). These mechanisms increased the core’s porosity and permeability (Figure 6 and Figure 7), improved fluid flow pathways, and enabled the mobilization of crude oil trapped in confined pore spaces. During this stage, *P. aeruginosa* exhibited lower biomass. Although it maintained the highest crude oil degradation capacity and could produce biosurfactant through biodegradation, the biosurfactant concentration could not be sustained at the levels observed in the initial culture medium. Furthermore, residual oil became distributed in inaccessible confined pore regions (Figure 5a,b), resulting in a significantly reduced WFR for *P. aeruginosa*. Although *B. licheniformis* also targets minerals, its biomass within the core and mineral dissolution capacity were inferior to those of *P. mucilaginosus*, resulting in limited improvements in porosity, permeability, and fluid mobility. Nevertheless, *B. licheniformis* achieved a 1.2% increase in WFR, which is larger than that noted for *P. aeruginosa* (0.9%).

### 3.4. MEOR Characteristics of Silicate Bacterium P. mucilaginosus

From the core traces of the microbial flooding pathways of the *P. mucilaginosus* culture medium, residual oil and remaining oil were observed at the inlet end, cross-section, and outlet end (Figure 5). At the inlet end, crude oil within macropores and their adjacent areas is driven toward the core interior, forming voids. Simultaneously, the non-porous outer surfaces of the core become fully coated with crude oil, which intimately bonds with mineral grains under displacement pressure (Figure 5a). At cross-sections, crude oil is exclusively distributed at pore throats, as oil can only penetrate into the core interior through the interconnected pore network (Figure 5b). Mineral dissolution and exfoliation have been observed on mineral grains within connected pores (Figure 5d,e). At the core outlet, filamentous crude oil displaced from connected pores has been observed (Figure 5c,f). Due to *P. mucilaginosus*’s deficiency in biosurfactant production, the crude oil fails to achieve effective emulsification, resulting in this filamentous morphology. EDX analyses across the three sampling planes reveal enhanced elemental diversity at the outlet (Figure 5a–c), which may serve as petrographic evidence of core mineral dissolution.

Compared with the culture media of *P. aeruginosa* and *B. licheniformis*, the culture medium of *P. mucilaginosus* has neither low surface tension nor low pH values, which makes the MEOR performance of *P. mucilaginosus* inferior to the other two at the microbial flooding stage. At this stage, the low surface tension of culture media is the main controlling factor of MFR. Therefore, the MFR of *P. mucilaginosus* is only 3.2%, which is primarily attributed to the improvement in the oil to water mobility ratio achieved with the initial culture medium. After entering the core, *P. mucilaginosus* is more likely to remain in the core than the other two. Therefore, its microbial cell concentration is higher during the water flooding stage. In the core, *P. mucilaginosus* degrades saturated hydrocarbons (1.9%, mainly n-alkanes of C10–C16) in crude oil to achieve proliferation, and its metabolites can act on minerals in a neutral pH environment. It changes the quantity and volume of pores, increases porosity (1.4%) and permeability (12.3 mD), and thus increases the WFR (3.7%).

Overall, the MR of *P. mucilaginosus* reaches 6.9%, which is lower than *P. aeruginosa* (7.9%), but their effective mechanisms are significantly different. *P. aeruginosa* can rapidly displace oil in a short time using the low surface tension of its culture medium at the microbial flooding stage, while *P. mucilaginosus* achieves its effects over a longer period by dissolving minerals in the core at the subsequent stage of water flooding. *B. licheniformis* relies on an acidic environment and limited biosurfactant content to achieve MR (4.8%). Regarding the mineral dissolution ability of *B. licheniformis*, it is stronger than that of *P. mucilaginosus* during the microbial flooding stage but weaker than that of *P. mucilaginosus* during the water flooding stage. Regarding the biosurfactant content of *B. licheniformis*, it remained consistently lower than that of *P. aeruginosa*. Therefore, their MFRs and WFRs also demonstrate the aforementioned characteristics. The unique feature of *P. mucilaginosus* lies in its highly efficient mineral dissolution and sustained effectiveness. In addition, *P. mucilaginosus* can continuously release mineral element ions, and this effect remains stable even after the WFR stops growing. These elemental ions can support microbial growth. Overall, *P. mucilaginosus* is suitable for injection at the beginning of crude oil development or combined with other microorganisms for application in MEOR.

## 4. Conclusions

To our best knowledge, it is the first report on experimental study of MEOR on low-permeability cores using the silicate bacterium *P. mucilaginosus*. Although the surface tension and pH value of the culture medium of *P. mucilaginosus* do not meet the requirements of traditional MEOR mechanisms for microorganisms, it improves the porosity and permeability of the core through two key capabilities: its ability to retain and proliferate extensively within the core and its long-term efficient dissolution of core minerals under neutral pH conditions. This ultimately achieves MR surpassing those of the acid-producing bacterium *B. licheniformis* and approaching the biosurfactant-producing bacterium *P. aeruginosa*. As a supplement to MEOR mechanisms and microbial selection, silicate bacteria can be applied during the initial water flooding stage of low-permeability reservoirs to improve porosity and permeability over the long term. They can also be combined with other microorganisms at any development stage to facilitate microbial survival. The MEOR functionality of silicate bacteria could be further explored to enhance the mobilization of corner-bound residual oil within reservoirs. Future research may reveal richer discoveries in MEOR using new mineral-targeting microorganisms like silicate bacteria.

## Figures and Tables

**Table 1:** The quantity and volume of pores in the cores before and after the experiment, as obtained using μCT data.

Core No.	Total Pore Quantity			Total Pore Volume *		
	Before (Pores)	After (Pores)	Difference (%)	Before (cm^3^)	After (cm^3^)	Difference (%)
C1	507,593	519,129	2.27	16.61	18.33	4.33
C4	539,832	510,014	−5.52	17.49	17.01	−2.74
C7	510,987	529,926	3.71	18.92	19.08	0.85

* The pore volume measured using μCT includes non-connected closed pores (Figure S3), which differs from the core saturation method described in Stage ①, thus leading to a larger pore volume compared to the values in Table S1.

## Data Availability

Data will be made available on request.

## Supplementary Materials

The following supporting information can be downloaded at: https://www.mdpi.com/article/10.3390/microorganisms13040738/s1, Figure S1: Microscope images of *Paenibacillus mucilaginosus* strain CICC20666 (left), *Pseudomonas aeruginosa* strain CICC10204 (middle) and *Bacillus licheniformis* strain CICC21886 (right) after 6 days of cultivation in the shaker with 120 rpm at 30 °C; Figure S2: The XIIC-YXLD core displacement device (Shengli Oilfield, Shandong, China) used in this experiment; Figure S3: μCT scan results were processed by Avizo to obtain the quantity and volume of core pores; Figure S4: Total ion current diagrams of saturated hydrocarbons in three experimental groups (*P. mucilaginosus*, *P. aeruginosa* and *B. licheniformis*); Figure S5: Total ion current diagrams of aromatic hydrocarbons in three experimental groups (*P. mucilaginosus*, *P. aeruginosa* and *B. licheniformis*); Figure S6: Content of saturated hydrocarbons in three experimental groups (*P. mucilaginosus*, *P. aeruginosa* and *B. licheniformis*); Figure S7: Content of aromatic hydrocarbons in three experimental groups (*P. mucilaginosus*, *P. aeruginosa* and *B. licheniformis*); Table S1: Basic parameters of nine artificial low-permeability cores used in the experiment; Table S2: Initial properties of culture media for *P. mucilaginosus*, *P. aeruginosa* and *B. licheniformis*; Table S3: Saturated hydrocarbons in crude oil detected by GC-MS; Table S4: Aromatic hydrocarbons in crude oil detected by GC-MS.
