# Supplementary material for: Lab-Scale Experimental Study of Microbial Enhanced Oil Recovery on Low-Permeability Cores Using the Silicate Bacterium Paenibacillus mucilaginosus
Source: Microorganisms. 2025 Mar 25;13(4):738. doi: 10.3390/microorganisms13040738 (PMC12029761; doi:10.3390/microorganisms13040738)
Supplement: Supplementary file 1 [file microorganisms-13-00738-s001.zip › microorganisms-3542065-supplementary.pdf]

Experimental study of microbial enhanced oil recovery on low permeability cores by silicate bacterium *Paenibacillus mucilaginosus*

Lei Li[a*], Chunhui Zhang[a], Peidong Su[a], Hongmei Mu[b]

[a]. School of Chemical & Environmental Engineering, China University of Mining & Technology (Beijing), Beijing 100083, PR China

[b]. State Key Joint Laboratory of Environment Simulation and Pollution Control, School of Environment, Tsinghua University, Beijing, 100084, PR China

* Correspondence: lileilunwen@126.com

Supplemental Materials

Table S1. Basic parameters of nine artificial low-permeability cores used in the experiment

Core No.	Diameter (mm)	Length (mm)	Porosity (%)	Permeability (mD)	PV (cm^3)	Experimental group
C1	25.11±0.03	110.24±0.15	15.93	36.9	8.7	*Paenibacillus. mucilaginosus*
C2	25.23±0.14	109.94±0.06	16.99	37.3	9.3	
C3	25.09±0.07	110.74±0.10	17.16	35.5	9.4	
C4	25.31±0.16	109.24±0.38	17.69	34.8	9.7	*Pseudomonas aeruginosa*
C5	25.54±0.07	107.30±0.19	15.98	33.0	8.8	
C6	25.42±0.28	114.74±0.15	16.45	37.1	9.6	
C7	25.11±0.39	110.09±0.29	16.97	35.8	9.2	*Bacillus licheniformis*
C8	24.91±0.30	116.94±0.51	17.50	36.3	10.0	
C9	25.01±0.21	112.64±0.03	16.72	33.2	9.2	

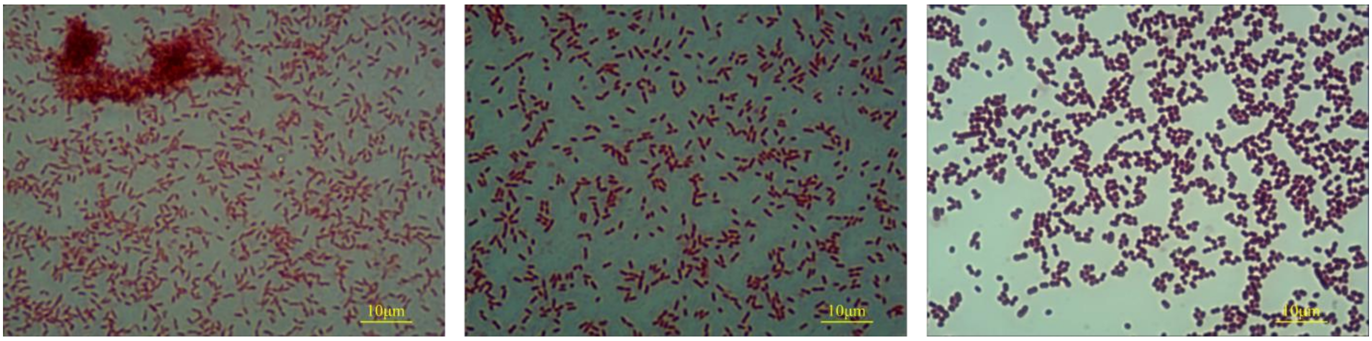

Figure S1. Microscope images of *Paenibacillus mucilaginosus* strain CICC20666 (left), *Pseudomonas aeruginosa* strain CICC10204 (middle) and *Bacillus licheniformis* strain CICC21886 (right) after 6 days of cultivation in the shaker with 120 rpm at 30°C.

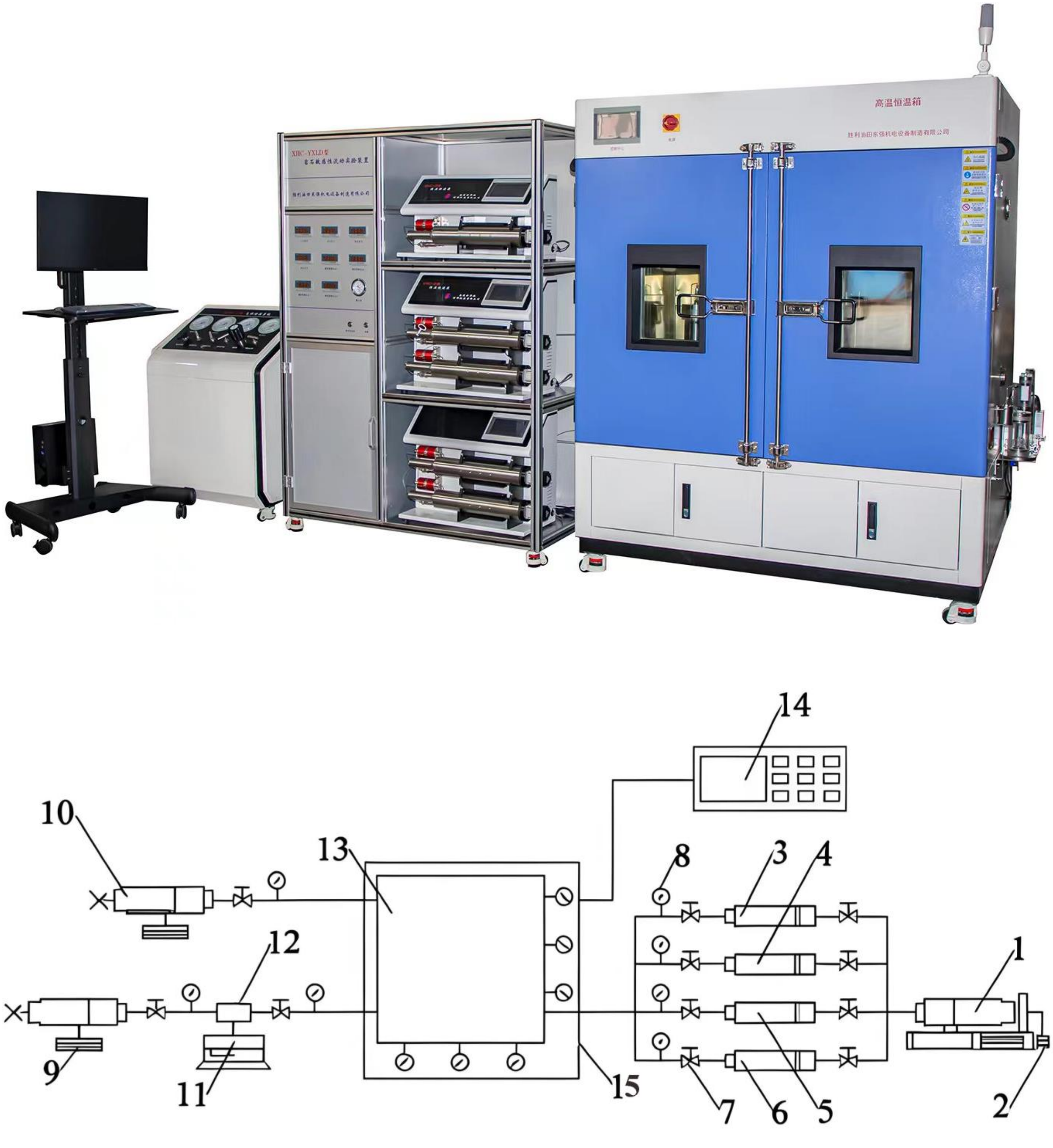

Figure S2. The XIIC-YXLD core displacement device (Shengli Oilfield, Shandong, China)

used in this experiment

Note: The image above is the physical object of the device, and the diagram below is the working schematic of the device. 1 denotes the drive pump, 2 denotes the drive pump storage tank, 3 denotes the deionized water piston container, 4 denotes the crude oil piston container, 5 denotes the microbial culture media piston container, 6 denotes the standby piston container, 7 denotes the regulating valve, 8 denotes the pressure gauge, 9 denotes the back pressure pump, 10 denotes the ring pressure pump, 11 denotes the flow metering device at outlet, 12 denotes the back pressure valve, 13 denotes the core holder, 14 denotes the temperature control device, 15 denotes the temperature control box.

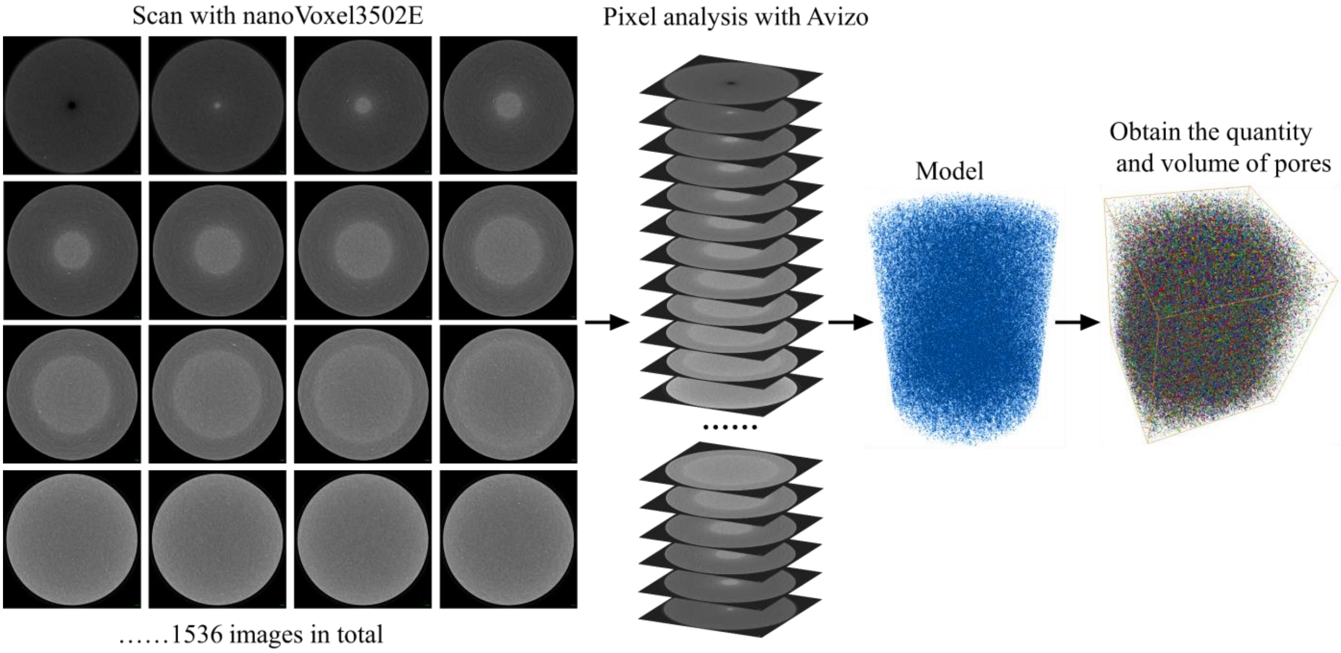

Figure S3. μCT scan results were processed by Avizo to obtain the quantity and volume of core pores

Table S2. Initial properties of culture media for *P. mucilaginosus*, *P. aeruginosa* and *B. licheniformis*

Microorganism	Cell concentration (cell/mL)	pH	Conductivity (μS/cm)	Redox potential (mV)	Surface tension (mN/m)	Viscosity (mPa·s)
P. mucilaginosus	10^8	6.3	2413	120	70.9	1.47

P. aeruginosa	7.1	2751	146	33.7	1.09
B. licheniformis	4.2	3187	164	60.8	1.15

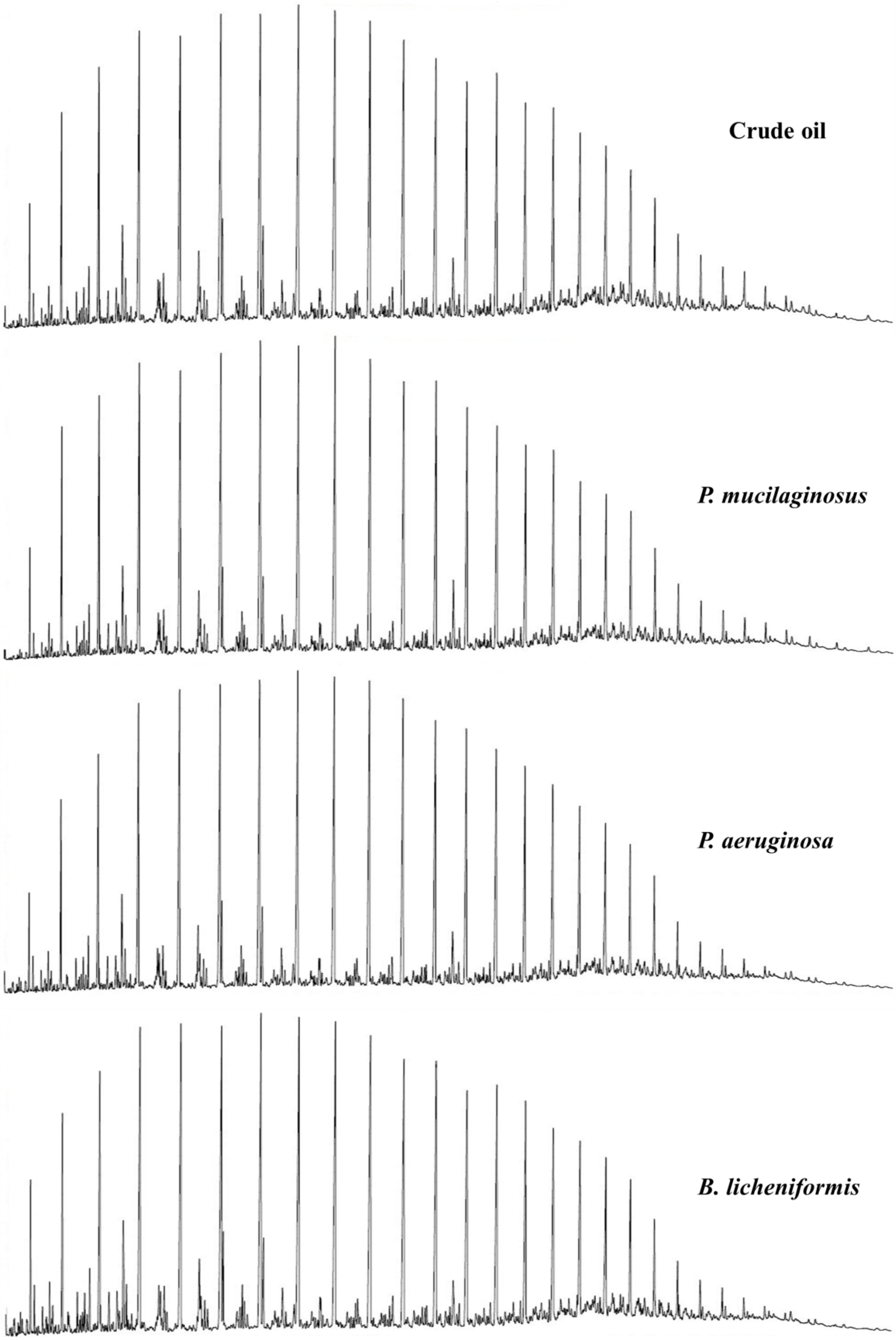

Figure S4. Total ion current diagrams of saturated hydrocarbons in three experimental groups (*P. mucilaginosus*, *P. aeruginosa* and *B. licheniformis*)

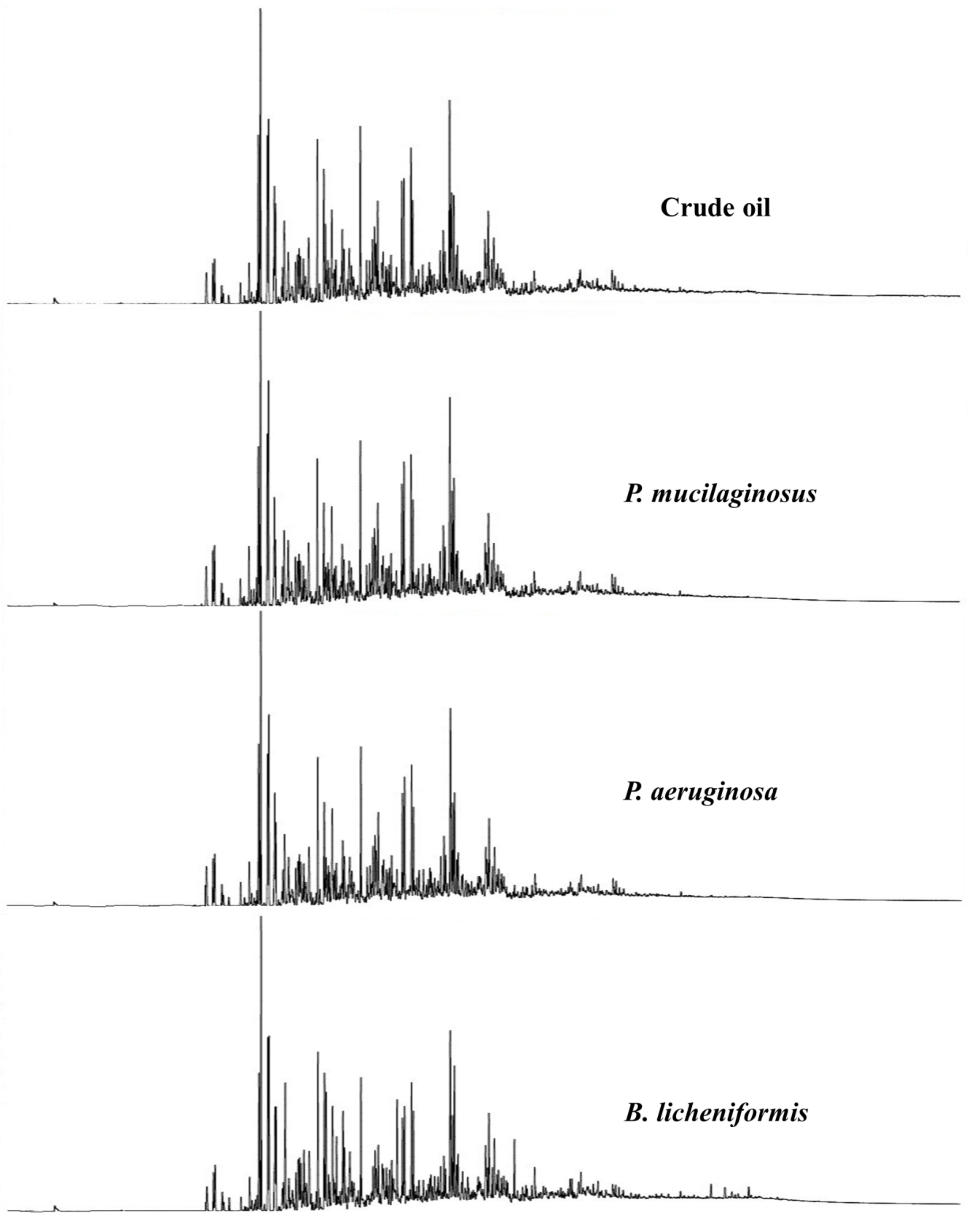

Figure S5. Total ion current diagrams of aromatic hydrocarbons in three experimental groups (*P. mucilaginosus*, *P. aeruginosa* and *B. licheniformis*)

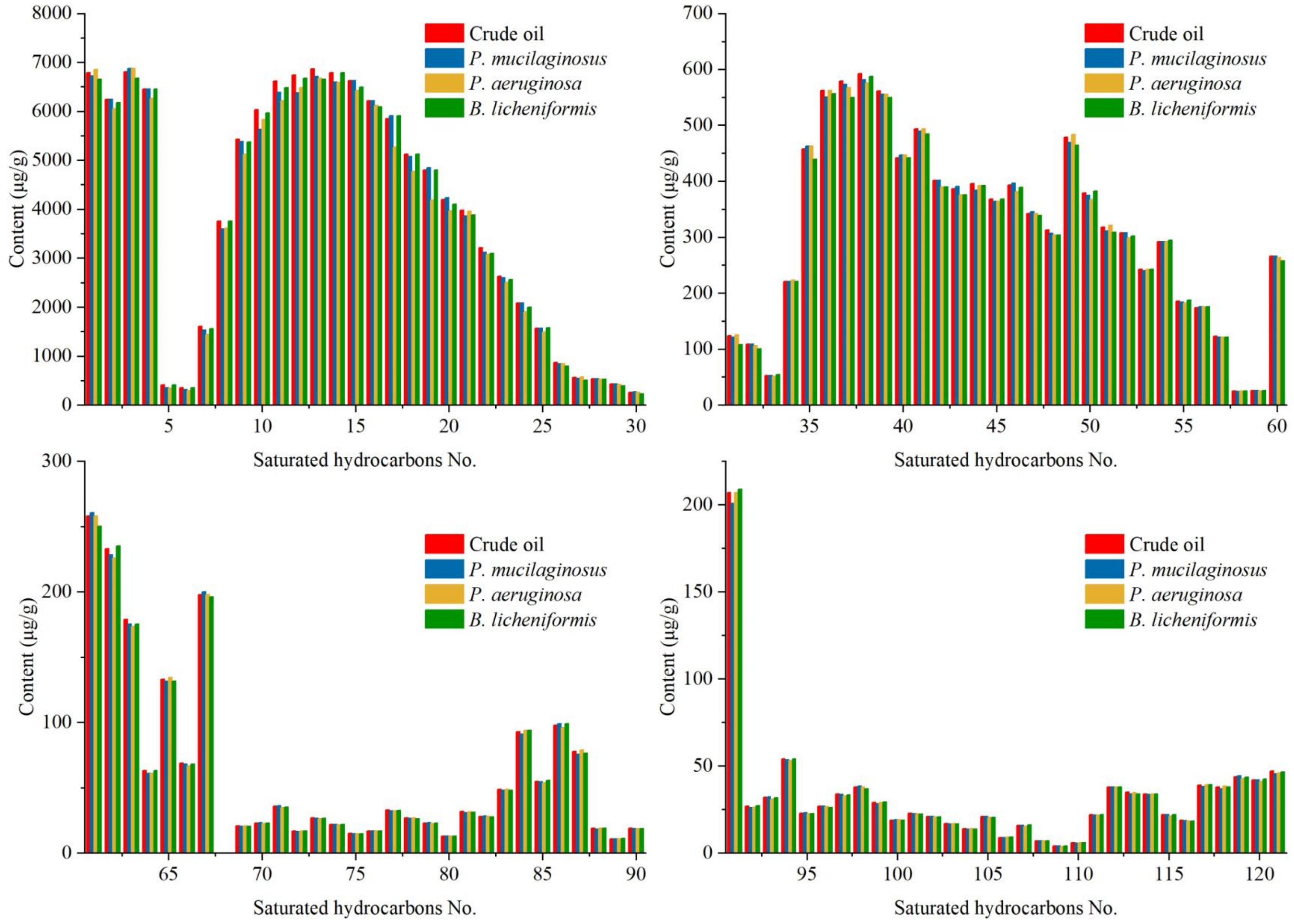

Figure S6. Content of saturated hydrocarbons in three experimental groups (*P. mucilaginosus*, *P. aeruginosa* and *B. licheniformis*)

Note: See Table S3 for the No. of saturated hydrocarbons

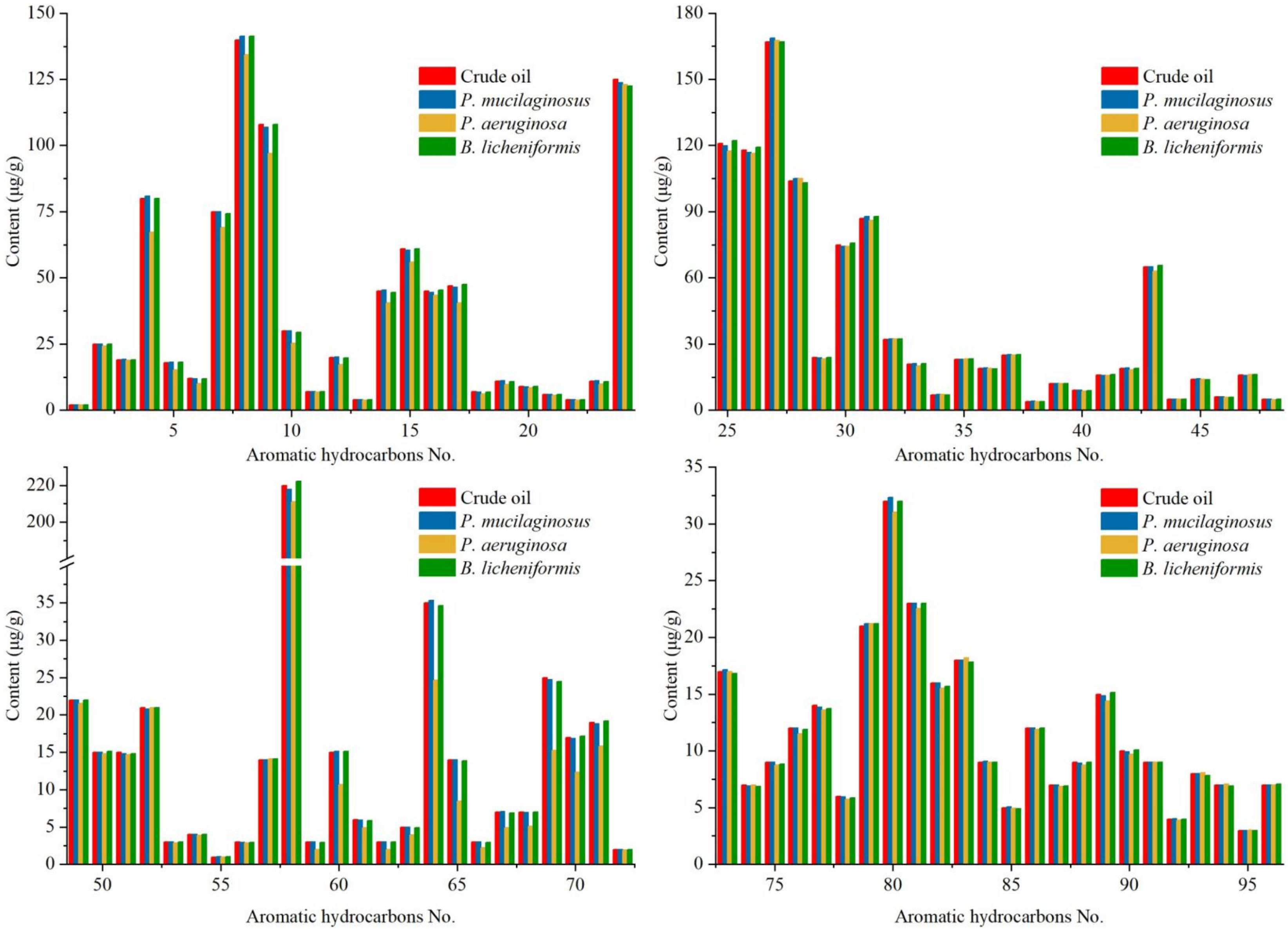

Figure S7. Content of aromatic hydrocarbons in three experimental groups (*P. mucilaginosus*, *P. aeruginosa* and *B. licheniformis*)

Note: See Table S4 for the No. of aromatic hydrocarbons

Table S3. Saturated hydrocarbons in crude oil detected by GC-MS

No.	Compound name	No.	Compound name	No.	Compound name
1	nC_{17}	2	Pr	3	nC_{18}
4	Ph	5	nC_{11}	6	nC_{12}
7	nC_{13}	8	nC_{14}	9	nC_{15}
10	nC_{16}	11	nC_{17}	12	nC_{18}
13	nC_{19}	14	nC_{20}	15	nC_{21}
16	nC_{22}	17	nC_{23}	18	nC_{24}
19	nC_{25}	20	nC_{26}	21	nC_{27}
22	nC_{28}	23	nC_{29}	24	nC_{30}
25	nC_{31}	26	nC_{32}	27	nC_{33}
28	nC_{34}	29	nC_{35}	30	nC_{36}
31	nC_{37}	32	nC_{38}	33	nC_{6}-alkyl cyclohexane
34	nC_{7}-alkyl cyclohexane	35	nC_{8}-alkyl cyclohexane	36	nC_{9}-alkyl cyclohexane
37	nC_{10}-alkyl cyclohexane	38	nC_{11}-alkyl cyclohexane	39	nC_{12}-alkyl cyclohexane
40	nC_{13}-alkyl cyclohexane	41	nC_{14}-alkyl cyclohexane	42	nC_{15}-alkyl cyclohexane
43	nC_{16}-alkyl cyclohexane	44	nC_{17}-alkyl cyclohexane	45	nC_{18}-alkyl cyclohexane
46	nC_{19}-alkyl cyclohexane	47	nC_{20}-alkyl cyclohexane	48	nC_{21}-alkyl cyclohexane
49	nC_{22}-alkyl cyclohexane	50	nC_{23}-alkyl cyclohexane	51	nC_{24}-alkyl cyclohexane
52	nC_{25}-alkyl cyclohexane	53	nC_{26}-alkyl cyclohexane	54	nC_{27}-alkyl cyclohexane
55	nC_{28}-alkyl cyclohexane	56	nC_{29}-alkyl cyclohexane	57	nC_{30}-alkyl cyclohexane
58	C_{14}-bicyclic sesquiterpene	59	C_{14}-bicyclic sesquiterpene	60	C_{15}-bicyclic sesquiterpene
61	C_{15}-bicyclic sesquiterpene	62	8β(H)-drimane	63	C_{15}-bicyclic sesquiterpene
64	C_{16}-bicyclic sesquiterpene	65	C_{15}-bicyclic sesquiterpene	66	C_{16}-bicyclic sesquiterpene
67	8β(H)-homodrimane	68	β-carotane	69	13β(H),14α(H)-C_{19}tricyclic terpane
70	13β(H),14α(H)-C_{20}tricyclic terpane	71	13β(H),14α(H)-C_{21}tricyclic	72	13β(H),14α(H)-C_{22}tricyclic terpane

			terpane		
73	13β(H),14α(H)-C_{23}tricyclic terpane	74	13β(H),14α(H)-C_{24}tricyclic terpane	75	13β(H),14α(H)-C_{25}tricyclic terpane(R)
76	13β(H),14α(H)-C_{25}tricyclic terpane(S)	77	C_{24}tetracyclic terpane	78	13β(H),14α(H)-C_{26}tricyclic terpane(R)
79	13β(H),14α(H)-C_{26}tricyclic terpane(S)	80	13β(H),14α(H)-C_{28}tricyclic terpane(R)	81	13β(H),14α(H)-C_{28}tricyclic terpane(S)
82	13β(H),14α(H)-C_{29}tricyclic terpane(R)	83	13β(H),14α(H)-C_{29}tricyclic terpane(S)	84	18α(H)-22,29,30-trisnorneohopane
85	17α(H)-22,29,30-trisnorhopane	86	17α,21β(H)-30-norhopane(C_{29}H)	87	18α,21β(H)-30-norneohopane(C_{29}Ts)
88	C_{30}dihopane	89	17β(H),21α(H)-30-normoretane	90	18α(H)-oleanane
91	17α(H),21β(H)-hopane(C_{30}H)	92	17β(H),21α(H)-moretane(C_{30}M)	93	17α(H),21β(H)-30-homohopane(22S)
94	17α(H),21β(H)-30-homohopane(22R)	95	gammacerane	96	17β(H),21α(H)-30-homomoretane(22S+22R)
97	17α(H),21β(H)-30,31-dihomohopane(22S)	98	17α(H),21β(H)-30,31-dihomohopane(22R)	99	17α(H),21β(H)-30,31,32-trihomohopane(22S)
100	17α(H),21β(H)-30,31,32-trihomohopane(22R)	101	17α(H),21β(H)-30,31,32,33-tetrahomohopane(22S)	102	17α(H),21β(H)-30,31,32,33-tetrahomohopane(22R)
103	17α(H),21β(H)-30,31,32,33,34-pentahomohopane(22S)	104	17α(H),21β(H)-30,31,32,33,34-pentahomohopane(22R)	105	C_{21}-5α(H)-pregnane
106	C22-5α(H)-homopregnane	107	13β(H),17α(H)-C_{27}diastrane(20S)	108	13β(H),17α(H)-C_{27}diastrane(20R)
109	13α(H),17β(H)-C_{27}diastrane(20S)	110	13α(H),17β(H)-C_{27}diastrane(20R)	111	5α(H),14α(H),17α(H)-sterane(20S)
112	5α(H),14β(H),17β(H)-sterane(20R)	113	5α(H),14β(H),17β(H)-sterane(20S)	114	24-methyl-5α(H),14α(H),17α(H)-sterane(20S)
115	24-methyl-5α(H),14β(H),17β(H)-sterane(20R)	116	24-methyl-5α(H),14β(H),17β(H)-sterane(20S)	117	24-methyl-5α(H),14α(H),17α(H)-sterane(20R)
118	24-ethyl-5α(H),14α(H),17α(H)-sterane(20S)	119	24-ethyl-5α(H),14β(H),17β(H)-sterane(20R)	120	24-ethyl-5α(H),14β(H),17β(H)-sterane(20S)
121	24-ethyl-5α(H),14α(H),17α(H)-sterane(20R)				

Table S4. Aromatic hydrocarbons in crude oil detected by GC-MS

No.	Compound name	No.	Compound name	No.	Compound name
1	naphthalene	2	2-methyl naphthalene	3	1-methyl naphthalene
4	1,6-dimethyl naphthalene	5	1,5-dimethyl naphthalene	6	1,2-dimethyl naphthalene
7	1,3,7-trimethyl naphthalene	8	1,3,6-trimethyl naphthalene	9	2,3,6-trimethyl naphthalene
10	1,2,6-trimethyl naphthalene	11	1,2,4-trimethyl naphthalene	12	1,2,5-trimethyl naphthalene
13	1,4,5-trimethyl naphthalene	14	1,3,5,7-tetramethyl naphthalene	15	1,3,6,7-tetramethyl naphthalene
16	2,3,6,7-tetramethyl naphthalene	17	1,2,6,7-tetramethyl naphthalene	18	1,2,3,7-tetramethyl naphthalene
19	1,2,3,6-tetramethyl naphthalene	20	1,2,4,6,7-pentamethyl naphthalene	21	1,2,3,5,7-pentamethyl naphthalene
22	1,2,3,6,7-pentamethyl naphthalene	23	1,2,3,5,6-pentamethyl naphthalene	24	phenanthrene
25	3-methyl phenanthrene	26	2-methyl phenanthrene	27	9-methyl phenanthrene
28	1-methyl phenanthrene	29	3-ethyl phenanthrene	30	1-ethyl phenanthrene
31	1,7-dimethyl phenanthrene	32	2,3-dimethyl phenanthrene	33	1,8-dimethyl phenanthrene
34	1,2-dimethyl phenanthrene	35	1,3,8-trimethyl phenanthrene	36	2,3,10-trimethyl phenanthrene
37	1,6,7-trimethyl phenanthrene	38	1,2,6-trimethyl phenanthrene	39	1,2,8-trimethyl phenanthrene
40	fluorene	41	3-methyl fluorene	42	2-methyl fluorene
43	1-methyl fluorene	44	4-methyl fluorene	45	dibenzothiophene
46	4-methyl dibenzothiophene	47	1-methyl dibenzothiophene	48	4-ethyl dibenzothiophene
49	4,6-dimethyl dibenzothiophene	50	2,4-dimethyl dibenzothiophene	51	2,6-dimethyl dibenzothiophene
52	3,6-dimethyl dibenzothiophene	53	2,8-dimethyl dibenzothiophene	54	1,7-dimethyl dibenzothiophene
55	1,2-dimethyl dibenzothiophene	56	dibenzofuran	57	4-methyl dibenzofuran
58	1-methyl dibenzofuran	59	biphenyl	60	3-methyl biphenyl
61	4-methyl biphenyl	62	3-ethyl biphenyl	63	3,5-dimethyl biphenyl
64	3,3'-dimethyl biphenyl	65	3,4'-dimethyl biphenyl	66	4,4'-dimethyl biphenyl
67	3,4-dimethyl biphenyl	68	3,5,3'-trimethyl biphenyl	69	3,5,4'-trimethyl biphenyl
70	3,4,3'-trimethyl biphenyl	71	3,4,4'-trimethyl biphenyl	72	fluoranthene
73	pyrene	74	benz[a]fluorene	75	benz[b]fluorene
76	2-methyl pyrene	77	4-methyl pyrene	78	1-methyl pyrene
79	benzo[a]anthracene	80	chrysene	81	3-methyl chrysene

82	2-methyl chrysene	83	4-methyl chrysene	84	6-methyl chrysene
85	1-methyl chrysene	86	benzo[b]fluoranthene	87	benzo[k]fluoranthene
88	benzo[e]pyrene	89	benzo[a]pyrene	90	perylene
91	C20 triaromatic steroid	92	C21 triaromatic steroid	93	C26 triaromatic steroid(20S)
94	C28 triaromatic steroid(20S)	95	C27 triaromatic steroid(20R)	96	C28 triaromatic steroid(20R)
